# Ecthyma gangrenosum caused by *Achromobacter xylosoxidans* bacteremia

**DOI:** 10.1590/0037-8682-0071-2023

**Published:** 2023-07-24

**Authors:** Sevil Alkan

**Affiliations:** 1 Çanakkale Onsekiz Mart University, Faculty of Medicine, Department of Infectious Diseases and Clinical Microbiology, Çanakkale,Turkey. Çanakkale Onsekiz Mart University Faculty of Medicine Department of Infectious Diseases and Clinical Microbiology Çanakkale Turkey

**FIGURE 1:**
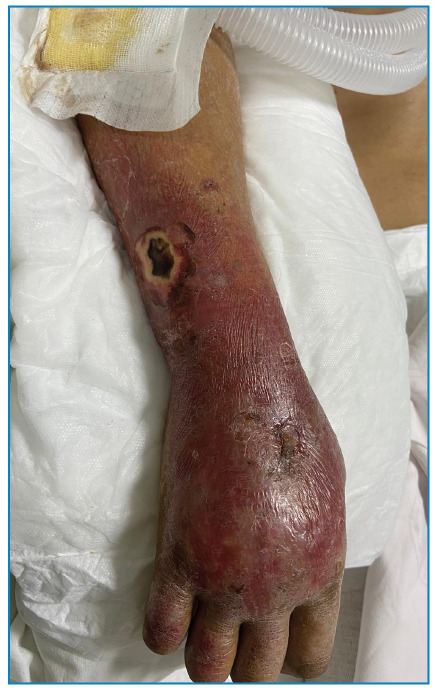
Gangrenous ulcer with black eschar on the forearm.

A 73-year-old woman with ovarian cancer undergoing chemotherapy presented with a three-day history of fever, vomiting, and confusion. She did not have a central venous access port. Physical examination revealed somnolence without meningeal irritation signs. A dry lesion located on the right forearm (Figure 1) was the only identifiable infectious focus. Neutropenia was not present. Blood and urine specimens were collected for culture, and piperacillin-tazobactam (3x4.5 g/day) was initiated. Urine culture yielded negative results, while two peripheral blood cultures grew *Achromobacter xylosoxidans*. Despite systemic antibiotic therapy, the patient succumbed to sepsis-related complications on the 5^th^ day of hospitalization. The cultured microorganism was sensitive to piperacillin-tazobactam. Histologic examination confirmed Ecthyma gangrenosum (EG). 

EG, a cutaneous infection, commonly affects immunocompromised patients with fulminant bacteremia. Dr. Lewellys Barker first described EG as a manifestation of *Pseudomonas aeruginosa* in 1897^1^. While *P. aeruginosa* remains the most commonly identified and implicated pathogen in EG, other microorganisms can also contribute to its etiology^2^. The primary site of EG is usually in the axillary and anogenital regions; however, cases with localized involvement of the arms, legs, trunk, and face have been documented in the literature^1^^,^^2^. EG has a poor prognosis, especially in neutropenic immunocompromised patients^1^. *A. xylosoxidans* bloodstream infections have been successfully treated with antibiotics like ceftazidime, piperacillin-tazobactam, carbapenems, and trimethoprim-sulfamethoxazole^3^.

This report presents a case of EG, a potentially fatal disease, and highlights the importance of prompt skin biopsies and microbiologic cultures for early diagnosis and treatment.
